# Extensin and Arabinogalactan-Protein Biosynthesis: Glycosyltransferases, Research Challenges, and Biosensors

**DOI:** 10.3389/fpls.2016.00814

**Published:** 2016-06-15

**Authors:** Allan M. Showalter, Debarati Basu

**Affiliations:** Department of Environmental and Plant Biology, Molecular and Cellular Biology Program, Ohio UniversityAthens, OH, USA

**Keywords:** biosynthesis, cell wall, extensin, arabinogalactan-protein, hydroxyproline, hydroxyproline-rich glycoproteins, glycosyltransferases, signaling

## Abstract

Recent research, mostly in *Arabidopsis thaliana*, has led to the identification and characterization of the glycosyltransferases responsible for the biosynthesis of two of the most functionally important and abundant families of plant cell wall proteins, extensins, and arabinogalactan-proteins. Extensin glycosylation involves monogalactosylation of serine residues by *O*-α-serine galactosyltransferase and the addition of oligoarabinosides one to five arabinose units in length to contiguous hydroxyproline residues by a set of specific arabinosyltransferase enzymes, which includes hydroxyproline *O*-β-arabinosyltransferases, β-1,2-arabinosyltransferases, and at least one α-1,3-arabinosyltransferase. AGP glycosylation, however, is much more complex and involves the addition of large arabinogalactan polysaccharide chains to non-contiguous hydroxyproline residues. These arabinogalactan chains are composed of β-1,3-galactan backbones decorated with β-1,6-galactose side chains that are further modified with α-arabinose as well as other sugars, including β-(methyl)glucuronic acid, α-rhamnose, and α-fucose. Specific sets of hydroxyproline *O*-β-galactosyltransferases, β-1,3-galactosyltransferases, β-1,6-galactosyltransferases, α-arabinosyltransferases, β-glucuronosyltransferases, α-rhamnosyltransferases, and α-fucosyltransferases are responsible for the synthesis of these complex structures. This mini-review summarizes the EXT and AGP glycosyltransferases identified and characterized to date along with corresponding genetic mutant data, which addresses the functional importance of EXT and AGP glycosylation. In one case, genetic mutant data indicate that the carbohydrate moiety of arabinogalactan-proteins may serve as an extracellular biosensor or signal for normal cellular growth. Finally, future research challenges with respect to understanding the function of these enzymes more completely and discovering and characterizing additional glycosyltransferases responsible for extensin and arabinogalactan-protein biosynthesis are also discussed.

## Introduction

Extensins (EXTs) and arabinogalactan-proteins (AGPs) are plant cell wall hydroxyproline-rich glycoproteins (HRGPs) and represent two of the most post-translationally modified and abundant protein families in plants and on Earth. EXTs are characterized by the repeating pentapeptide sequence Ser-Hyp-Hyp-Hyp-Hyp (SO_4_) or variations thereof, such as SO_3_ and SO_5_ as well as by monogalactosylation of some Ser residues and oligoarabinosylation of most Hyp residues (Showalter, 1993; Kieliszewski and Lamport, 1994; Liu et al., 2016). EXTs are rod-like glycoproteins that exist in a polyproline II helix stabilized by the oligoarabinosides wrapping around the protein backbone (Van Holst and Varner, 1984; Shpak et al., 2001). Some EXTs contain Tyr residues that are modified to form intramolecular isodityrosine crosslinks or intermolecular di-isodityrosine or pulcherosine crosslinks (Brady and Fry, 1997; Brady et al., 1998). Intramolecular crosslinks cause “kinks” in the rods, while intermolecular crosslinks result in formation of EXT networks in the wall. Such crosslinks may be important in regulating growth and development and in forming a defensive barrier to pathogens. EXT-pectin crosslinks also exist, but their extent and functional significance is unknown (Qi et al., 1995).

In contrast, AGPs are much more heavily glycosylated than EXTs and are not diagnostically characterized by SO_3_, SO_4_, and SO_5_ repeats. Instead, AGPs have an abundance of Hyp, Ala, Ser, and Thr residues and are often characterized by the occurrence of Ala-Pro, Pro-Ala, Ser-Pro, Thr-Pro, Val-Pro, and Gly-Pro dipeptide repeats. Extensive glycosylation of AGPs occurs on clustered, non-contiguous Hyp residues and consists of arabinose and galactose-rich polysaccharide side chains. Arabinogalactan (AG) side chains attached to the AGP core protein allow AGPs to react with a chemical called Yariv reagent, which is useful for identifying, quantitating, and localizing AGPs (Showalter, 2001; Seifert and Roberts, 2007). AG polysaccharides are composed of β-1,3-galactan backbones decorated with β-1,6-galactose side chains that are further modified with α-arabinose as well as other sugars, including β-(methyl)glucuronic acid, α-rhamnose, and α-fucose. Based on microscopic observations and modeling of Hyp-AG side chains, AGPs are spheroidal or rod-like molecules, corresponding to the wattle blossom and twisted hairy rope models of AGP structure, respectively. While there is little evidence for AGP-AGP crosslinking, there is evidence for AGP-pectin and AGP-pectin-arabinoxylan crosslinking (Kjellbom et al., 1997; Tan et al., 2013). Most AGPs have glycosylphosphatidylinositol (GPI) anchors, which allow them to attach to the plasma membrane and reside in the periplasm. Specific phospholipases are thought to allow for release of these AGPs into the wall.

**FIGURE 1:**
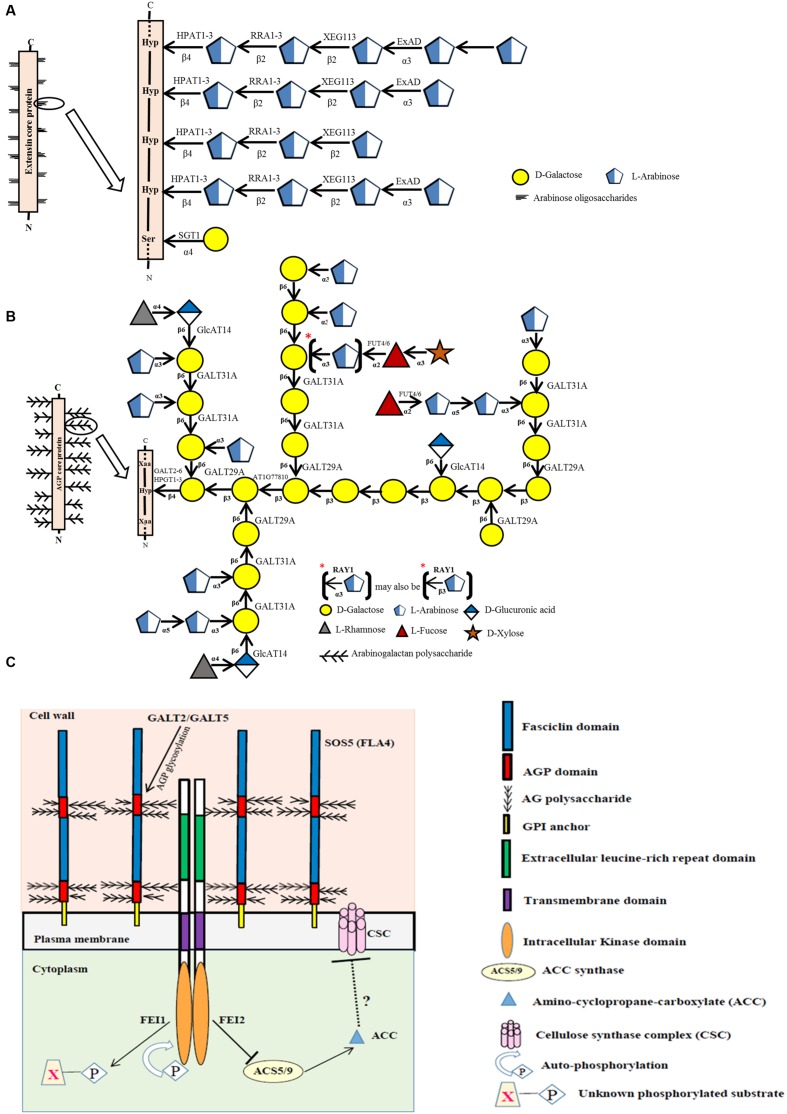
**Biosynthesis of EXTs (A) and AGPs (B) and the possible role of a fasciclin-like AGP (SOS5/FLA4) as an extracellular biosensor (C). (A)** Sites of action of known glycosyltransferases acting on EXTs are depicted within a representative EXT glycomodule sequence found in an EXT molecule. **(B)** Sites of action of known glycosyltransferases acting on AGPs are depicted within a representative AGP glycomodule sequence found within an AGP molecule. **(C)** Proposed model that links AGP-Hyp GALTs, GALT2 and GALT5 with SOS5 (FLA4) and FEI1/FEI2 in regulating cellular signaling of root growth. Signaling of normal root growth likely involves GALT2/GALT5-dependent glycosylation of SOS5 and glycosylated SOS5 binding FEI1/FEI2, which in turn triggers binding of FEI1/FEI2 to ACC synthase, specifically ACS5, to form a scaffold complex. The ACC synthase in the scaffold is thought to lead to the localized production of ACC, a potential signaling molecule of cell wall integrity. In contrast, when GALT2/GALT5-dependent SOS5 glycosylation is inhibited or when SOS5 or FEI1/FEI2 is disrupted, ACC synthase (ACS5) is not bound to FEI1/FEI2 and thus is not part of the scaffold. Consequently, the unbound ACC synthase produces non-localized ACC, which is converted to ethylene, and serves to inhibit cellulose synthase/cellulose biosynthesis. Such weaker or compromised cell walls lead to root tip swelling in response to elevated NaCl or sucrose treatment. It is also likely that GALT3, GALT4, and GALT6 as well as HPGT1, HPGT2, and HPGT3 play redundant or partially redundant roles to GALT2 and GALT5 in this pathway.

Much of the recent work on EXTs and AGPs has focused on the biosynthesis of their carbohydrate moieties and the functional contributions of these moieties (Tan et al., 2012; Knoch et al., 2014). Most of this work has used *Arabidopsis thaliana* as the model. Interestingly, cellulosic biofuel research, which in part requires a deeper understanding of cell wall biosynthesis and the associated biosynthetic enzymes, has served as a major driving force to examine HRGP biosynthesis and particularly the associated glycosyltransferases (GTs) responsible for decorating their protein backbones with specific sugar side chains. This mini-review focuses on these GTs that act on EXTs (**Figure 1A**) and AGPs (**Figure 1B**), the use of reverse genetics/mutants to dissect the functional roles of these sugar moieties (**Table 1**), and research challenges in this field.

**Table 1:** Information on the enzymes, genes, and genetic mutants for EXT^∗^ and AGP^∗∗^ glycosylation.

Enzyme Abbreviation	Enzyme	GT Family	Localization	Gene Identifier	Mutants	Mutant phenotypes	Reference
SGT1^∗^	serine *O*-α-galactosyltransferase	GT96	ER and Golgi	*At3g01720*	*sgt1-1* (SALK_059879) *sgt1-2* (SALK_054682)	Longer roots, larger rosettes, and reduced SGT activity	Saito et al., 2014; Velasquez et al., 2015

HPAT1^∗^ HPAT2^∗^ HPAT3^∗^	hydroxyproline *O*-β-arabinosyltransferase	GT95 GT95 GT95	Golgi	*At5g25265* *At2g25260* *At5g13500*	*hpat1* (GABI_298B03), *hpat2* (SAIL_178_H04), *hpat3* (SALK_047668)	Impaired pollen tube growth, early senescence, early flowering, defects in cell wall thickening, enhanced hypocotyl elongation, shorter root hairs, and reduction in HPAT activity	Ogawa-Ohnishi et al., 2013; Velasquez et al., 2015; MacAlister et al., 2016

RRA1^∗^ RRA2^∗^ RRA3^∗^	β-1,2-arabinosyltransferase	GT77 GT77 GT77	Golgi Golgi Golgi	*At1g75120* *At1g75110* *At1g19360*	*rra1* (SAIL_590_G09 Garlic_76_G04), *rra2* (Garlic_244_A03 SAIL_70_D08) *rra3* (GABI_233B05)	Reduced root hair growth and reduced levels of arabinose in the mutant	Egelund et al., 2007; Velasquez et al., 2011, 2015

XEG113^∗^	β-1,2-arabinosyltransferase	GT77	Golgi	*At2g35610*	*xeg113–1* (SALK_066991), *xeg113–3* (SALK_058092)	Reduced root hair growth and reduced levels of arabinose in the mutant	Gille et al., 2009; Velasquez et al., 2011

ExAD^∗^	α-1,3-arabinosyltransferase	GT47	–	–	–	–	Velasquez et al., 2012

GALT2^∗∗^ GALT3^∗∗^ GALT4^∗∗^ GALT5^∗∗^ GALT6^∗∗^ HPGT1^∗∗^ HPGT2^∗∗^ HPGT3^∗∗^	hydroxyproline-*O*-β-galactosyltransferase	GT31 GT31 GT31 GT31 GT31 GT31 GT31 GT31	ER and Golgi Golgi Golgi Golgi Golgi Golgi – –	*At4g21060* *At3g06440* *At1g27120* *At1g74800* *At5g62620* *At5g53340* *At4g32120* *At2g25300*	*galt2-1* (SALK_117233) *galt2-2* (SALK_141126) *galt3-1* (SALK_085633) *galt3-2* (SALK_005178) *galt4-1* (SALK_136251) *galt4-2* (SALK_131723) *galt5-1* (SALK_105404) *galt5-2* (SALK_115741) *galt6-1* (SAIL_59_D08) *galt6-2* (SAIL_70_B02) *hpgt1-1* (SALK_007547) *hpgt2-1* (SALK_070368) *hpgt3-1* (SALK_009405)	Reduced root hair length and density for *galt2*, *galt3*, and galt5, reduced seed set for *galt4* and *galt6*, reduced adherent seed coat mucilage for *galt3* and *galt6*, premature senescence for *galt6*, root and pollen tubes are less sensitive to β-Yariv reagent for *galt2-6*, reduced root growth and root tip swelling in elevated NaCl and sucrose for *galt2-6*, reduced AGP content and Hyp-*O*-GALT activity for *galt2-6*; *galt2galt5* double mutants show more severe and pleiotropic phenotypes than *galt* single mutants with respect to reduced root hair length and density, adherent seed coat mucilage, AGP content, and Hyp-*O*-GALT activity; *galt2galt5* double mutants also display more rosette leaves, delayed flowering, and shorter siliques Both the double *(hpgt2-1hpgt3-1)* and triple *(hpgt1-1hpgt2-1hpgt3-1)* mutants exhibit longer lateral roots, increased root hair length and density, thicker roots, smaller rosette leaves, shorter petioles, shorter inflorescence stems, reduced fertility in the lower portion of the inflorescence, and shorter siliques, reduced AGP content and Hyp-*O*-GALT activity	Basu et al., 2013, 2015a,b, 2016; Ogawa-Ohnishi and Matsubayashi, 2015

AT1G77810^∗∗^	β-1,3-galactosyltransferase	GT31	Golgi	*At1g77810*	Not reported	–	Qu et al., 2008

GALT31A^∗∗^	β-1,6-galactosyltransferase	GT31	Golgi	*At1g32930*	*galt31A* (FLAG_379B06)	Embryo lethal mutant	Geshi et al., 2013
GALT29A^∗∗^	β-1,6-galactosyltransferase	GT29	Golgi	*At1g08280*	Not reported	–	Dilokpimol et al., 2014

GlcAT14A^∗∗^ GlcAT14B^∗∗^ GlcAT14C^∗∗^	β-1,6-glucuronosyl transferase	GT14 GT14 GT14	Golgi – –	*At5g39990* *At5g15050* *At2g37585*	*glcat14a-1* (SALK_06433) *glcat14a-2* (SALK_043905)	Enhanced cell elongation in seedlings and reduced GlcA substitution on β–1,6–galactobiose and β–1,3–galactan in their AGPs	Knoch et al., 2013; Dilokpimol and Geshi, 2014

FUT4^∗∗^ FUT6^∗∗^	α-1,2-fucosyltransferase	GT37 GT37	– Golgi	*At2g15390* *At1g14080*	*fut4-1* (SAIL_284_B) *fut4-2* (SALK_12530) *fut6-1* (SALK_0783) *fut6-2* (SALK_09950)	Reduced root growth under salt stress, *fut4* lacks fucose only in leaf AGPs whereas *fut4fut6* double mutants lack fucose both in leaf and root AGPs	Wu et al., 2010; Liang et al., 2013; Tryfona et al., 2014

RAY1^∗∗^	β-arabinofuranosyl transferase	GT77	Golgi	*At1g70630*	*ray1-1* (SALK_053158) *ray1-2* (GABI_001C09)	Reduced root growth, reduced rosette size, and delayed inflorescence size, decreased arabinose in etiolated seedlings, roots, and rosette leaves	Gille et al., 2013

*^∗^Denotes an enzyme that acts on EXTs. ^∗∗^Denotes an enzyme that acts on AGPs.*

## EXT Glycosyltransferases

At least nine genes are involved with EXT glycosylation in *Arabidopsis* (**Figure 1A**; **Table 1**). One of these genes, *SERINE GALACTOSYLTRANSFERASE1* (*SGT1*), is responsible for adding single galactose residues to Ser residues in SO_4_ sequences, but not in SP_4_ sequences. This gene was identified in *Chlamydomonas reinhardtii* by sequencing a protein with *O*-α-serine galactosyltransferase activity; subsequently, homologous genes were identified in *Nicotiana tabacum* and *Arabidopsis thaliana* (Saito et al., 2014). The SGT1 enzyme was localized primarily to the endoplasmic reticulum (ER) membrane and to the Golgi, and its activity requires the presence of Hyp residues. SGT1 was initially absent from the CAZy database, but now is considered a member of the GT96 family.

The other eight genes involved with EXT glycosylation encode arabinosyltransferases with specific substrate specificities and enzymatic activities. Three of these genes (*HPAT1*, *HPAT2*, and *HPAT3*) encode hydroxyproline *O*-β-arabinosyltransferases (HPATs), which add single arabinose residues to Hyp residues in SO_4_ sequences (Ogawa-Ohnishi et al., 2013). These genes were identified in *Arabidopsis* by affinity purification and sequencing proteins that demonstrated HPAT activity. These three genes/enzymes are related to GT8 family members, but were placed in a new family, GT95. Three other genes, *REDUCED RESIDUAL ARABINOSE1-3* (*RRA1*, *RRA2*, and *RRA3*), encode arabinosyltransferases that add the second arabinose residue in a β-1,2 linkage (Egelund et al., 2007; Velasquez et al., 2011). These three genes/enzymes are GT77 family members and were discovered by genetic mutant analysis. Another gene, *XYLOGLUCANASE113* (*XEG113*), encodes the arabinosyltransferase which adds the third arabinose residue also in a β-1,2 linkage (Gille et al., 2009). This gene/enzyme is also in GT77 and was discovered from a genetic mutant analysis involving treatment of *Arabidopsis* with a fungal endogluconase. Another gene, *EXTENSIN ARABINOSE DEFICIENT* (*ExAD*), encodes the arabinosyltransferase that adds the fourth arabinose in an α-1,3 linkage (Velasquez et al., 2012). This gene/enzyme is a member of GT47 and was discovered using a genetic mutant approach similar to that used to find the *RRAs*. All the EXT arabinosyltransferases identified above are localized to the Golgi.

While the extent of Hyp-arabinosylation is variable for EXTs with most having three or four arabinose residues per Hyp, some EXTs have five arabinose residues. This indicates that at least one more arabinosyltransferase gene likely exists to encode the transfer of this fifth arabinose.

## EXT Glycosyltransferase Mutants

EXT GT genetic mutants have provided a powerful approach to determine the functional contributions of the carbohydrate moieties that decorate EXTs. The current list of these mutants appears in **Table 1** along with their respective mutant phenotypes. For example, *sgt1* mutants display larger rosettes and longer roots (Saito et al., 2014; Velasquez et al., 2015). The *hpat* mutants demonstrate several pleiotropic phenotypes including impaired pollen tube growth, early senescence, early flowering, defects in cell wall thickening, enhanced hypocotyl elongation, and shorter root hairs (Ogawa-Ohnishi et al., 2013; Velasquez et al., 2015; MacAlister et al., 2016). Interestingly, *rra* and *xeg113* mutants also share one of the phenotypes displayed by *hpat* mutants, namely reduced root hair growth (Egelund et al., 2007; Gille et al., 2009; Velasquez et al., 2011, 2015).

## AGP Glycosyltransferases

To date, 17 different genes corresponding to seven distinct enzymes are involved with AGP glycosylation in *Arabidopsis* (**Figure 1B**; **Table 1**). AGP glycosylation is initiated by the action of hydroxyproline *O*-β-galactosyltransferase, which places the first galactose residue onto Hyp residues in AGP core proteins. Eight genes encoding this activity are known. Five of these were identified using bioinformatics and verified by heterologous expression coupled with an *in vitro* enzyme assay and by genetic mutant analysis (Basu et al., 2013, 2015a,b). These genes, named *GALT2*, *GALT3*, *GALT4*, *GALT5*, and *GALT6*, exist as a small gene family within GT31. Each gene encodes a GALT domain as well as a GALECTIN domain. Another similar gene in GT31, *GALT1*, also encodes both of these domains, but is not involved in AGP glycosylation; instead this gene is involved in adding galactose to Lewis a structures (Strasser et al., 2007). The other three genes were found by sequencing proteins selected by affinity chromatography with an AGP peptide and by heterologous expression coupled with an enzyme assay and by genetic mutant analysis (Ogawa-Ohnishi and Matsubayashi, 2015). These genes, named *HPGT1*, *HPGT2*, and *HPGT3*, form another small gene family within GT31, but lack a GALECTIN domain. GALT3-6 and HPGT1 are localized to Golgi; however, GALT2 is localized to both ER and Golgi, indicating AGP glycosylation may begin in the ER, but likely mainly occurs in Golgi.

Three additional AGP galactosyltransferase genes are known. One encodes a β–1,3-galactosyltransferase activity and likely functions in β–1,3-galactan backbone synthesis. This gene, *At1g77810*, was identified by bioinformatics and demonstrated to add galactose to a synthetic β–1,3-galactose disaccharide following heterologous expression in COS cells (Qu et al., 2008). This gene resides in GT31 along with a number of other putative *Arabidopsis* galactosyltransferases that may act on AGPs. Another GT31 gene, *GALT31A*, encodes a β–1,6-galactosyltransferase (Geshi et al., 2013). This gene was heterologously expressed in *E. coli* and *Nicotiana benthamiana* and elongated β–1,6-galactan side chains of AGP glycans. The other characterized galactosyltransferase gene, *GALT29A*, demonstrated the same enzymatic activity encoded by *GALT31A* as well as an additional branching activity, adding β–1,6-galactose to β–1,3-galactans of AGP glycans (Dilokpimol et al., 2014). *GALT29A* was identified as being co-expressed with *GALT31A* and resides in GT29, not GT31. GALT29A and GALT31A interact with one another based on fluorescence resonance energy transfer analysis and demonstrated enhanced enzymatic activity, suggesting these enzymes act cooperatively and exist in an enzyme complex (Dilokpimol et al., 2014).

Three β-glucuronosyltransferase genes, *GlcAT14A*, *GlcAT14B*, and *GlcAT14C*, add glucuronic acid to AGPs (Knoch et al., 2013; Dilokpimol and Geshi, 2014). All three genes/enzymes are members of GT14 and are reported to add glucuronic acid to both β–1,6- and β–1,3-galactose chains in an *in vitro* enzyme assay following heterologous expression in *Pichia pastoris*. Glucuronic acid imparts a negative charge to AGPs and provides a potential site for calcium binding; both of these properties likely have functional ramifications (Lamport and Várnai, 2013).

Two α-fucosyltransferase genes, *FUT4* and *FUT6*, encode enzymes which add α-1,2-fucose residues to AGPs (Wu et al., 2010; Liang et al., 2013; Tryfona et al., 2014). These genes were identified by bioinformatics and demonstrated to add fucose to AGPs following heterologous expression in BY2 cells (Wu et al., 2010). These enzymes appear to be partially redundant as they display somewhat different AGP substrate specificities. Both genes/enzymes reside in GT37. Fucose is not present in all AGPs, including those produced by BY2 cells, a fact that was exploited to verify their activity.

Finally, one gene encodes an arabinosyltransferase that may act on AGPs based on genetic mutant analysis in *Arabidopsis*. Mutations in this gene resulted in plants with a reduced level of arabinose, particularly 3-linked arabinofuranose, in their AGPs, leading to its name, *REDUCED ARABINOSE YARIV1*, or *RAY1* (Gille et al., 2013). This gene/enzyme is in GT77, the same family that contains RRA1-3 AND XEG113, enzymes responsible for EXT arabinosylation (Egelund et al., 2007; Gille et al., 2009; Velasquez et al., 2011). Thus, it was not surprising when heterologous expression of *RAY1* in *Nicotiana benthamiana* demonstrated β–arabinosyltransferase activity to methyl β–galactose. Since α-linked arabinose, and not β-linked arabinose, is reported in AGPs, it remains unclear whether *RAY1* functions in AGP biosynthesis.

## AGP Glycosyltransferase Mutants

Many AGP GT genetic mutants are characterized and beginning to reveal the importance of AGP glycosylation to AGP function (**Table 1**). The *galt2-6* single mutants revealed some physiological phenotypes under normal growth conditions, including reduced root hair length and density for *galt2*, *galt3*, and *galt5*, reduced seed set for *galt4* and *galt6*, reduced adherent seed coat mucilage for *galt3* and *galt6*, and premature senescence for *galt6* (Basu et al., 2013, 2015a,b). However, *galt2galt5* double mutants showed more severe and pleiotropic physiological phenotypes than the single mutants with respect to root hair length and density and seed coat mucilage; double mutants also displayed more rosette leaves, delayed flowering, and shorter siliques (Basu et al., 2013, 2015a,b). Such findings are consistent with the idea that as additional redundant or partially redundant genes are knocked-out for a particular enzyme activity, more severe physiological phenotypes will be revealed. The pleiotropic nature of these mutations highlights the widespread occurrence of AGPs throughout the plant and the functional importance of AGP carbohydrate moieties. These ideas were also supported by *hpgt* mutant data. Specifically, *hpgt1-3* single mutants showed no obvious phenotypes under normal growth conditions, however, *hpgt1hpgt2hpgt3* triple mutants showed several pleiotropic phenotypes including longer lateral roots, increased root hair length and density, thicker roots, smaller rosette leaves, shorter petioles, shorter inflorescence stems, reduced fertility, and shorter siliques (Ogawa-Ohnishi and Matsubayashi, 2015). Some of these phenotypes were observed in the *galt* mutants.

With respect to other AGP galactosyltransferase genes, a single knockout mutant only exists for *GALT31A* (Geshi et al., 2013). This mutant, however, is embryo lethal, exhibiting abnormal cell division in the hypophysis, despite specific expression of *GALT31A* in the embryo suspensor cells.

Knockout mutants for *GlcAT14A*-*C* showed enhanced cell elongation rates in dark grown hypocotyls and light grown roots during seedling growth (Knoch et al., 2013; Dilokpimol and Geshi, 2014). However, since several sugars were altered in these mutants in addition to reduced glucuronic acid, the mutant phenotypes may reflect contributions of these other Knockout mutants for *FUT4* and *FUT6* show no obvious phenotypes under normal conditions; however, root growth is significantly inhibited in these mutants when grown in salt (Liang et al., 2013; Tryfona et al., 2014). This conditional phenotype reveals the importance of this minor sugar component of some AGPs with respect to root growth and salt sensitivity.

Finally, a knockout mutant for *RAY1* exhibits pleiotropic effects, reduced root growth, reduced rosette size, and reduced inflorescence size (Gille et al., 2013). The above ground mutant phenotypes, however, may be a secondary effect of impaired root growth.

Clearly, a range of phenotypes is observed for these AGP GT mutants. This indicates the important and diverse functions that AGP glycans play in plant vegetative and reproduction growth and development.

## Research Challenges

As research on EXT and AGP GTs moves forward, several questions and challenges come to mind:

(1) There is a need to identify and characterize the remaining EXT and AGP GT genes/enzymes in *Arabidopsis*, including the EXT arabinosyltransferase for the fifth arabinose and other AGP β–1,3-galactosyltransferases and β–1,6-galactosyltransferases along with many α-arabinosyltransferases and α-rhamnosyltransferases, which await discovery. There is also a need to extend this research to other plant species. Interestingly, there is no evidence for any processive GTs involved in synthesizing HRGPs, such as the β–1,3-galactan backbone or β–1,6-galactan side chains of AGPs. Thus, it is conceivable that each sugar in an AGP (or EXT) is added by a specific, non-processive enzyme.
(2) It is important to determine the exact enzymatic activity for each EXT and AGP GT. In other words, does the GT act on all or only a subset of EXTs or AGPs? What is the peptide or glycan substrate specificity for each EXT or AGP GT? How do these GTs regulate the extent, length, sequence, and heterogeneity of the sugar additions?
(3) Clearly, multiple EXT and AGP GTs exist and catalyze identical, or very similar, enzymatic activities. Thus, it becomes important to determine whether these genes are redundant, partially redundant, or non-redundant. Such determinations are likely to involve examination of organ/tissue-specific expression patterns, elucidating substrate specificities, and genetic mutant analysis.
(4) For some AGP hydroxyproline *O*-β-galactosyltransferases, namely GALT2-6, what is the function of the GALECTIN domain?
(5) Do EXT and AGP GTs act cooperatively and in enzyme complexes? To date, only one study has provided support for this idea (Dilokpimol et al., 2014).
(6) For the GT mutants, it becomes important to know exactly which EXTs and AGPs are modified and the chemical details of such modifications in order to relate structure to function.
(7) It will be useful to grow and examine the various GT mutants side-by-side under identical environmental conditions, ideally in the same laboratory, to ensure accurate comparisons.
(8) Higher order GT mutants, particularly in cases where gene families encode a particular EXT GT (e.g., *RRA1–3*) or AGP GT (e.g., *GALT2–6*, *HPGT1–3*), should be produced and examined for functional redundancy.

## AGPs as Extracellular Sensors or Signals

Based on bioinformatic predictions, approximately half of the AGPs in *Arabidopsis* are GPI-anchored to the outer leaflet of the plasma membrane (Showalter et al., 2010). This cell surface location provides an ideal venue for GPI-anchored AGPs to sense the extracellular environment and relay such information to other plasma membrane proteins involved in cellular signaling. For example, GPI-anchored AGPs may act as biochemical pressure/turgor sensors or may serve to selectively bind and release calcium ions (Lamport and Várnai, 2013). In recent years, evidence for a GPI-anchored AGP named SALT OVERLY SENSITIVE 5 (SOS5) being involved in cellular signaling of root growth has accumulated (Shi et al., 2003; Xu et al., 2008). SOS5, also known as FASCICLIN-LIKE AGP4 or FLA4, acts in a single genetic pathway that involves two cell wall leucine-rich, repeat receptor-like kinases (RLKs), called FEI1 and FEI2. Recently, GALT2 and GALT5 were shown to exist in this genetic pathway, and likely are responsible for glycosylating SOS5 to allow it to function in this signaling pathway by directly or indirectly interacting with FEI1/FEI2 (**Figure 1C**) (Basu et al., 2016). GALT3, GALT4, and GALT6 as well as HPGT1-3, may play redundant or partially redundant roles to GALT2 and GALT5 in this pathway. While the downstream mechanism remains to be elucidated, cellulose synthesis is the likely target, which leads to weaker cell walls and root tip swelling in the mutants.

GALT2, GALT5, SOS5, and FEI2 also function together in another signaling pathway leading to seed coat mucilage adherence (Basu et al., 2016). Mutations in these four genes, as well as higher order mutant combinations, lead to reduced mucilage adherence. While the downstream mechanism here may also involve cellulose synthesis, it is suggested to primarily involve pectin structure or assembly (Harpaz-Saad et al., 2011, 2012; Griffiths et al., 2014).

## Concluding Remarks

Carbohydrate largely defines the molecular surfaces and hence the functions of EXTs and AGPs. Thus, it is of importance to understand how the various sugars are added to EXTs and AGPs and contribute to their functions not only in terms of basic science, but also for potential applications in biomimetics and biofuel production. Genetic mutant analysis of the various GT mutants is beginning to provide such insight. Finally, up to this point, GTs involved with EXT and AGP biosynthesis were identified and characterized mainly from *Arabidopsis*; however, future work on isolating and characterizing homologous or similar GTs acting on EXTs and AGPs from other members of the plant kingdom promises to extend and enrich our knowledge of these important enzymes.

## Author Contributions

AS outlined and wrote the manuscript. DB assisted with writing the manuscript and prepared the figure and table for the manuscript.

## Conflict of Interest Statement

The authors declare that the research was conducted in the absence of any commercial or financial relationships that could be construed as a potential conflict of interest.

## Abbreviations

EXTs: Extensins
GALT: Galactosyltransferase
GT: Glycosyltransferase
Hyp: Hydroxyproline
Ser: Serine
